# Evaluating the metabolic effects of neoadjuvant treatment in clear cell renal cell carcinoma using hyperpolarized [1-^13^ C]pyruvate MRI

**DOI:** 10.1007/s00261-025-05313-z

**Published:** 2025-12-10

**Authors:** Ines Horvat-Menih, Mary A McLean, Jonathan Birchall, Maria Jesus Zamora Morales, Marta Wylot, Stephan Ursprung, Ramona Woitek, Eva Serrao, Ashley Grimmer, Elizabeth Latimer, Alixander S Khan, Andrew N Priest, Andrew B Gill, Joshua D Kaggie, Martin J Graves, Tristan Barrett, James MS Wason, Helen Mossop, Martin Thomas, Sulekha Said, Anne Y Warren, Kate Fife, Tim Eisen, Athena Matakidou, Will Ince, Brent O’Carrigan, James O Jones, Sarah J Welsh, Thomas J Mitchell, James N Armitage, Antony CP Riddick, Grant D Stewart, Ferdia A Gallagher

**Affiliations:** 1 Department of Radiology, University of Cambridge, Cambridge, United Kingdom; 2 Netherlands Cancer Institute, Amsterdam, Netherlands; 3 Research Center for Medical Image Analysis and Artificial Intelligence, Danube Private University, Krems, Austria; 4 Cambridge University Hospitals NHS Foundation Trust, Cambridge, United Kingdom; 5 Department of Radiology, University of Cambridge, Cambridge, United Kingdom; 6 Population Health Sciences Institute, Newcastle University, Newcastle upon Tyne, United Kingdom; 7 Pinto Medical Consultancy, Cambridge, United Kingdom; 8 Department of Surgery, University of Cambridge, Cambridge, United Kingdom; 9 Department of Surgery, University of Cambridge, Cambridge, United Kingdom; 10 Cambridge University Hospitals NHS Foundation Trust, Cambridge, United Kingdom

**Keywords:** Neoadjuvant treatment, Clear cell renal cell carcinoma, Hyperpolarized [1-^13^C]pyruvate MRI, Treatment response monitoring

## Abstract

**Abstract:**

Despite recent therapeutic advances, renal cell carcinoma (RCC) has a high mortality rate. The development of new neoadjuvant strategies requires reliable companion biomarkers of early and successful response to treatment. A change in tumor size is a late measure of response and novel targeted imaging-based biomarkers may be more accurate for treatment response prediction. Here we evaluated the potential of hyperpolarized carbon-13 MRI (HP ^13^C-MRI), following the injection of hyperpolarized ^13^C-pyruvate, to assess response to neoadjuvant treatment in four patients with clear cell RCC as an exploratory outcome within a prospective clinical trial. The change in the tumor lactate-to-pyruvate ratio (LAC/PYR) following treatment varied across the patients: mean percentage change ± S.D. = +6 ± 27%. The largest decrease in LAC/PYR ratio was in the patient treated with cediranib monotherapy (-21%), followed by a smaller reduction in a patient receiving the combination of cediranib and olaparib (-14%). An increase in the LAC/PYR ratio post-treatment was observed in the second patient receiving combination treatment (+ 21%), with the largest increase in the patient receiving olaparib monotherapy (+ 35%). Metabolic changes were observed following treatment in the absence of significant changes in tumor size. In summary, HP ^13^C-MRI successfully captured heterogeneous metabolic responses to cediranib and olaparib therapy, revealing both increases and decreases in tumor lactate labelling, independent of any morphologic change. Finally, this is the first study to evaluate the potential of clinical HP ^13^C-MRI to assess early treatment response in renal cancer by using a range of therapeutics.

**Key take home message:**

The study provides preliminary evidence supporting HP ^13^C-MRI as a promising imaging biomarker for evaluating early metabolic changes in renal cell carcinoma following neoadjuvant therapy.

**Clinical Trials Registry:**

NCT03741426, Registration date: 13 November 2018.

**Supplementary Information:**

The online version contains supplementary material available at 10.1007/s00261-025-05313-z.

## Introduction

Renal cell carcinoma (RCC) is the most common kidney malignancy with an increasing incidence globally, but with no decline in mortality rates [1]. Surgery with curative intent is the treatment of choice for clinically-fit patients whose disease burden is amenable to resection, yet ~ 30% experience recurrence [1, 2]. Neoadjuvant approaches allow rapid treatment of patients with aggressive localized disease whilst potentially minimising the need for adjuvant therapy after surgery [3].

A particular challenge for studies evaluating neoadjuvant therapy is the lack of validated predictive biomarkers in RCC, which delays the advancement of novel agents [3]. Currently treatment response is determined using the RECIST 1.1 criteria (Response Evaluation Criteria in Solid Tumors), which are based on the change in size of target lesions [4]. However, there is a lack of evidence for the use of RECIST-defined progression as a clinically-validated endpoint for treatment modification, and morphologic changes alone may be insufficient for capturing response to novel targeted therapies [4]. Therefore, there is an unmet clinical need to develop non-invasive approaches which accurately detect changes in tumour biology that can be harnessed to assess clinical response to therapy.

Hyperpolarised [1-^13^ C]pyruvate MRI (HP ^13^C-MRI) is a novel clinical imaging technique to probe metabolic conversion of injected hyperpolarised [1-^13^ C]pyruvate to [1-^13^ C]lactate. A decrease in tumor lactate labelling has been demonstrated after several weeks of treatment in prostate cancer [5], and the technique has been used to detect response to neoadjuvant therapy in breast cancer after only 7–11 days, which outperformed conventional MRI in distinguishing pathological complete response from non-responders [6]. In renal cancer, HP ^13^C-MRI has shown potential for assessing tumor aggressiveness, but has not yet been probed as a marker of treatment response [7, 8].

The WIRE study (WIndow-of-opportunity clinical trial platform for evaluation of novel treatment strategies in REnal cell cancer) is a phase II, multi-arm, non-randomised trial which probes the biological mechanism of novel targeted therapies in clear cell RCC (ccRCC) during the interval between diagnosis and surgery (NCT03741426). Here we report an exploratory outcome within the WIRE study assessing the application of HP ^13^C-MRI to probe metabolic treatment response to: cediranib (a tyrosine kinase inhibitor), olaparib (a poly ADP-ribose polymerase or PARP inhibitor), and both agents in combination. The metabolic MRI findings were compared to changes in tumor size, perfusion, and diffusion.

## Results

Following the Methods as detailed in the Supplementary file, four patients with a diagnosis of resectable ccRCC who were recruited to the WIRE trial underwent successful HP ^13^C-MRI before and after treatment. In brief, the patient was imaged in a 3 T MRI (MR750, GE Healthcare, Waukesha, WI, USA) equipped with a ^13^C-transmit clamshell coil and a ^13^C-tuned 8-channel array coil (Rapid Biomedical, Rimpar, Germany) positioned over the tumor. A 250 mM hyperpolarized pyruvate solution (0.4 mL/kg) was administered via a power injector (Medrad) at 5 mL/s. ^13^C images were acquired starting 12 s after the injection, with a temporal resolution of 4 s for 20 time points, and had a true resolution of 17 × 17 mm.

An overview of the patient clinical characteristics is presented in Table 1.

**Table 1:** Patient characteristics

	Age at detection [years] Sex	Tumor diameter at detection	Presentation at detection (relevant comorbidities); ECOG status	Treatment (days) Days between pre- and post-treatment MRI	Preoperative RECIST 1.1	Stage and grade of the tumor
Patient 1	48y, M	9.2 cm	Right flank pain (hypertension); ECOG 0	Cediranib (25 days) 30 days	Stable disease	ypT3a pN0 M0; Grade 4
Patient 2	61y, F	11.5 cm	Hematuria (cachexia, anemia); ECOG 1	Cediranib + olaparib (26 days) 29 days	Stable disease	ypT3b N0 M1 (lungs); Grade 4
Patient 3	58y, M	9.2 cm	Incidental (hypertension); ECOG 0	Cediranib + olaparib (22 days) 21 days	Stable disease	ypT3a pN0 M0; Grade 3
Patient 4	75y, M	6.5 cm	Weight loss (hypertension, pulmonary embolism); ECOG 1	Olaparib (28 days) 35 days	Stable disease	ypT3b pN0 M0; Grade 3

**Fig. 1:**
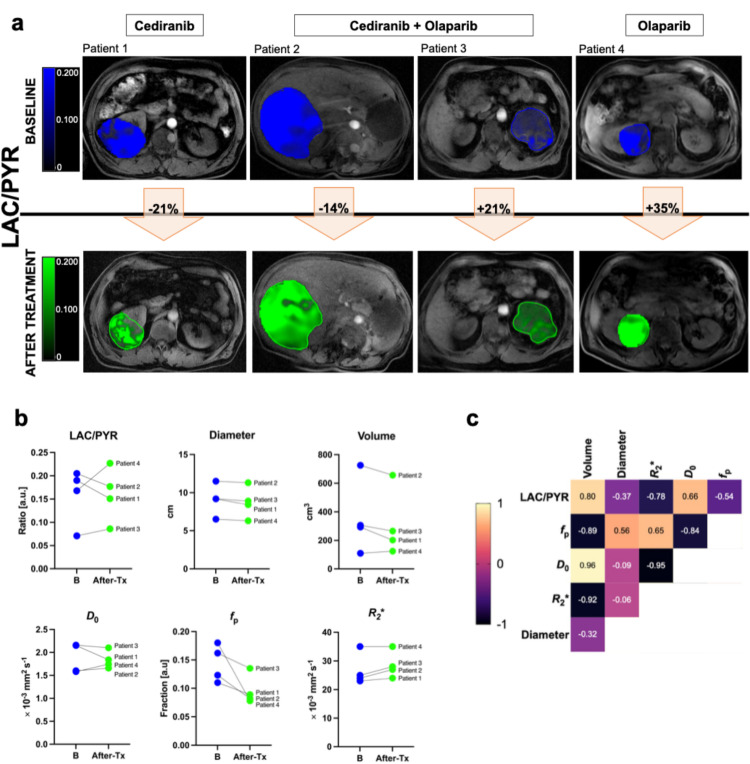
**a** Representative LAC/PYR maps of the four patients overlaid on T_1_w images, with tumor ROIs annotated in blue (baseline) and green (post-treatment). Percentage changes between the baseline and post-treatment LAC/PYR ratios are presented with arrows for each individual patient. ROI = region of interest. **b** Paired plots representing changes in the imaging parameters within tumor ROIs pre- (blue) and post-treatment (green). The LAC/PYR ratio and *D*_0_ showed variable changes, whereas *f*_p_ and *R*_2_* were most consistently altered post-treatment. **c** Correlation analyses between these imaging parameters are presented in a heatmap, labelled with respective Pearson coefficients. B = baseline, After-Tx = after-treatment

Representative LAC/PYR ratio maps overlaid on anatomical T_1_w images before and after treatment are presented in Fig. 1a. Quantitative results are presented in Suppl. Table S1. The mean LAC/PYR ratio change for all four patients showed a slight increase of + 6% post-treatment but with a significant interpatient variation in S.D. of 27%. Patients 1 (cedirinib monotherapy) and 2 (cediranib and olaparib) demonstrated a decrease in the tumor LAC/PYR of −21% (baseline: 0.19; post-treatment: 0.15) and − 14% (baseline: 0.21; post-treatment: 0.18) respectively. Patients 3 (cediranib and olaparib) and 4 (olaparib monotherapy) showed an increase in the tumor LAC/PYR ratio post-treatment of + 21% (baseline: 0.07; post-treatment: 0.09) and + 35% (baseline: 0.17; post-treatment: 0.23) respectively.

The perfusion surrogate, *f*_p_, showed the greatest and most consistent changes post-treatment of −31 ± 18%, followed by the hypoxia surrogate, *R*_2_*, of + 18 ± 21% (Fig. 1b). The treatment effect on the diffusion coefficient (*D*_0_) was variable, with Patients 1 and 3 exhibited a decrease (−14% and − 3%, respectively), and Patients 2 and 4 an increase (+ 4% and + 10%, respectively). Only small alterations were observed in tumor diameters, with a mean decrease of −4% from 9.1 cm at baseline to 8.7 cm post-treatment (S.D. 3%), and the RECIST 1.1 indicating stable disease in all patients. Mean volumetric changes were greater: −10% over the treatment time course (S.D. 18%); Patient 4 (olaparib only) showed a 13% increase in tumor volume post-treatment which was associated with the greatest increase in *D*_0_ of + 10% indicating reduced restriction, the largest decrease in the *f*_p_ of −57%, as well as the highest baseline *R*_2_*, which remained high post-treatment (0% change). Correlative analysis as shown in Fig. 1c, with detailed results in Suppl. Table S2, confirmed a significant relationship between the %change in tumor volume and *D*_0_ (Pearson *r* = 0.96, *P* < 0.05), and though non-significant, a strong negative correlation coefficient with *f*_p_ (Pearson *r* = −0.84, *P* = 0.165) and *R*_2_* (Pearson *r* = −0.92, *P* = 0.052). Importantly, the LAC/PYR changes were independent of the alterations in other imaging metrics.

## Discussion

Four patients undergoing neoadjuvant treatment showed variable changes in the tumor LAC/PYR ratio, which occurred in the absence of significant changes in diameter, RECIST 1.1 outcome, or volume, suggesting that HP ^13^C-MRI may distinguish early metabolic alterations before morphological changes can be measured. These results are similar to those reported previously in breast cancer, where an increase in LAC/PYR ratio predicted the response to standard-of-care neoadjuvant chemotherapy after 7–11 days [6].

We have demonstrated previously that the LAC/PYR ratio increased in breast cancer patients undergoing olaparib therapy, irrespective of their response [6]. PARP inhibitors are known to increase intracellular concentrations of nicotinamide adenine dinucleotide (NAD+), an essential co-factor for many redox pathways, which then becomes available as a co-factor for the reaction catalyzed by lactate dehydrogenase, promoting the exchange between pyruvate and lactate [9]. An increase in lactate labelling could therefore represent drug engagement with the target. The LAC/PYR ratio decreased in the patient receiving cediranib monotherapy (−21%) and increased in the patient receiving olaparib alone (+ 35%), with the two patients on combinational therapy showing values between these two values: this supports the suggestion that cediranib and olaparib have opposing effects on tumor metabolism, as well as the potential for distinguishing between responders and non-responders in patients receiving olaparib. Furthermore, Patient 4 (olaparib monotherapy) displayed the greatest decrease in the perfusion fraction (−57%) across all the patients, even though olaparib is not an antiangiogenic agent, and this may be related to increased tumor hypoxia, as measured by the hypoxia surrogate *R*_2_*, which has been previously suggested to enhance the action of olaparib [10]. Finally, a significant positive correlation between volume changes and *D*_0_ was found. While more data is needed to fully elucidate the biological mechanism behind this correlation following treatment, this successful response could induce necrosis, which increases *D*_0_ due to alterations in extracellular fluid, and may in turn alter the tumor size. However, this response is likely to be highly specific to an individual therapeutic agent and its mechanism of action [11].

The present study is the first to evaluate the potential of HP ^13^C-MRI in assessing early treatment response in renal cancer. HP ^13^C-MRI successfully detected metabolic changes following treatment in the absence of significant changes in size on clinical proton MRI, demonstrating the feasibility of the novel technique. Larger trials are needed to confirm these findings and to correlate these changes with long-term clinical outcome.

## Supplementary Information

Below is the link to the electronic supplementary material.

Supplementary material 1 (DOCX 33.7 kb)

## Data Availability

Data availability: Data generated or analyzed during the study are available from the corresponding author upon request.Code availability: Custom computer code was used to generate the results for imaging and is available upon request.

## Abbreviations

AUC: Area under the curve
ccRCC: Clear cell renal cell carcinoma
DWI: Diffusion-weighted imaging
D0: Diffusion coefficient
ECOG: Eastern Cooperative Oncology Group
FOV: Field of view
fp: Perfusion fraction
HP 13C-MRI: Hyperpolarised carbon-13 MRI
IVIM: Intravoxel incoherent motion
LAC/PYR: Lactate-to-pyruvate ratio
NAD+: Nicotinamide adenine dinucleotide
PARP: Poly ADP-ribose polymerase
RCC: Renal cell carcinoma
RECIST: Response Evaluation Criteria in Solid Tumors
ROI: Region of interest
S.D.: Standard deviation
SNR: Signal-to-noise ratio
TNM: Tumor, Node, Metastasis
WHO/ISUP: World Health Organisation/International Society for Urologic Pathologists
WIRE: WIndow-of-opportunity clinical trial platform for evaluation of novel treatment strategies in REnal cell cancer
